# Dr. John H. Tanner

**Published:** 1906-05

**Authors:** 


Dr. John H. Tanner, a graduate of the University of Buffalo, 1863, formerly of Spencer, N. Y., died at his home at Harford, N. Y., March 12, 1906, aged 70 years. His death was caused by a self-inflicted gunshot wound in the head.

